# Gastrointestinal stromal tumour in Meckel's diverticulum

**DOI:** 10.1186/1477-7819-5-50

**Published:** 2007-05-12

**Authors:** K Chandramohan, Mudit Agarwal, Gopal Gurjar, Rohan C Gatti, Mahesh H Patel, Preeti Trivedi, Kiran C Kothari

**Affiliations:** 1 Department of Surgical Oncology, Gujarat Cancer and Research Centre, Ahmedabad, Gujarat, India; 2 Department of Pathology, Gujarat Cancer and Research Centre, Ahmedabad, Gujarat, India; 3 Division of Surgical Oncology, Regional Cancer Centre, Trivandrum, Kerala, India

## Abstract

**Background:**

Meckel's Diverticulum is the most commonly encountered congenital anomaly of the small intestine, occurring in approximately 2% of the population. Occasionally Meckel's diverticulum harbors neoplasms.

**Case presentation:**

A 65 year old gentleman, presented with a pelvic mass. On exploratory laparotomy, it turned out to be gastrointestinal stromal tumour (GIST) arising from Meckel's diverticulum. Short history and review of literature are discussed.

**Conclusion:**

Neoplasms occurring from Meckel's diverticulum, even though rare, should be considered as differential diagnosis of pelvic masses arising from bowel, wherever imaging modalities fail to give a definitive diagnosis.

## Background

Meckel's diverticulum, the most commonly encountered congenital anomaly of the small intestine, affects 2% of the population [1,2]. The vast majority of Meckel's diverticulae are incidentally discovered during autopsy, laparotomy, or barium studies [3]. Meckel's diverticulum is surgically removed only when a complication arises or a neoplasia develops. The tumors are infrequent and observed only in 0.5–3.2% of the Meckel's diverticula. Of these, 12% tumors are GIST. We are reporting one such incidence, where we came across a Meckel's diverticulum harboring GIST.

## Case presentation

**Figure 1:**
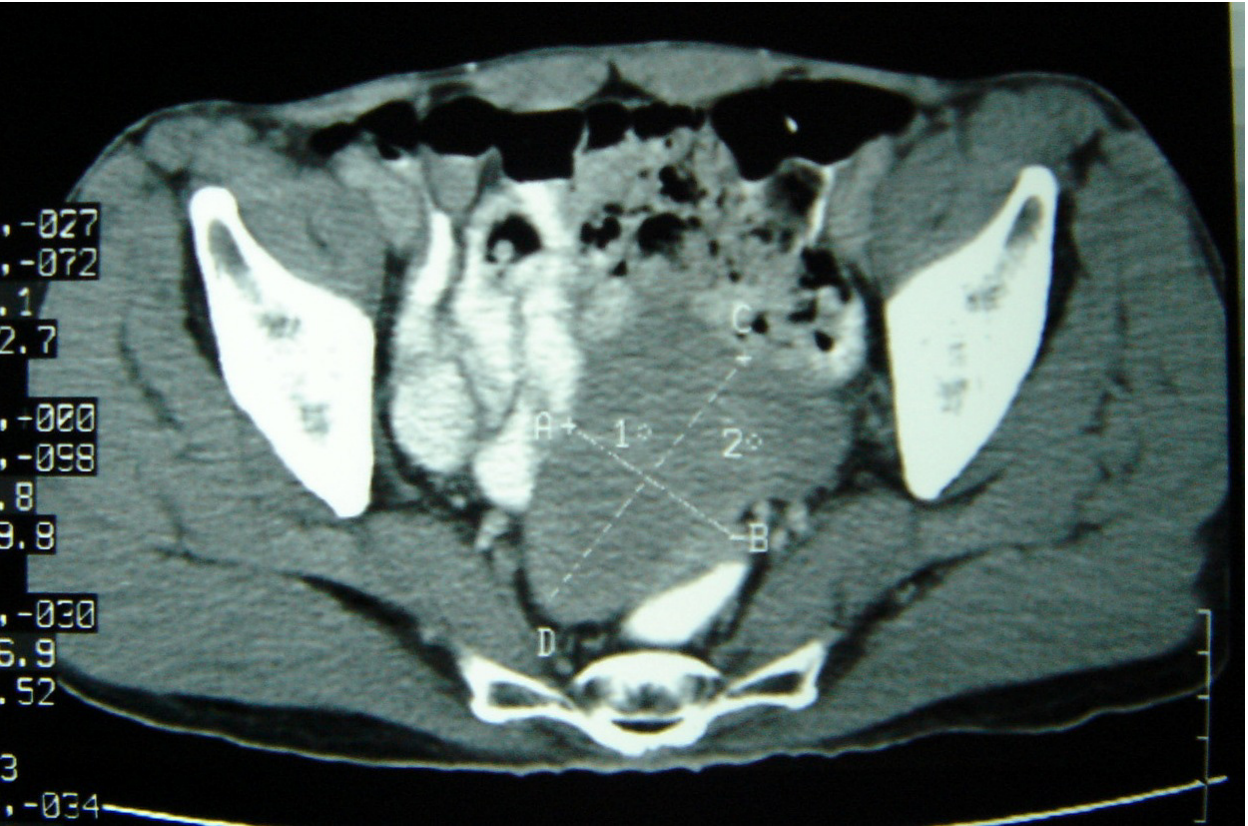
Preoperative CT scan showing pelvic mass; Contrast enhanced CT scan showing lobulated mass lesion in pelvis compressing anterior wall of sigmoid colon and located posteriosuperior to the urinary bladder.

**Figure 2:**
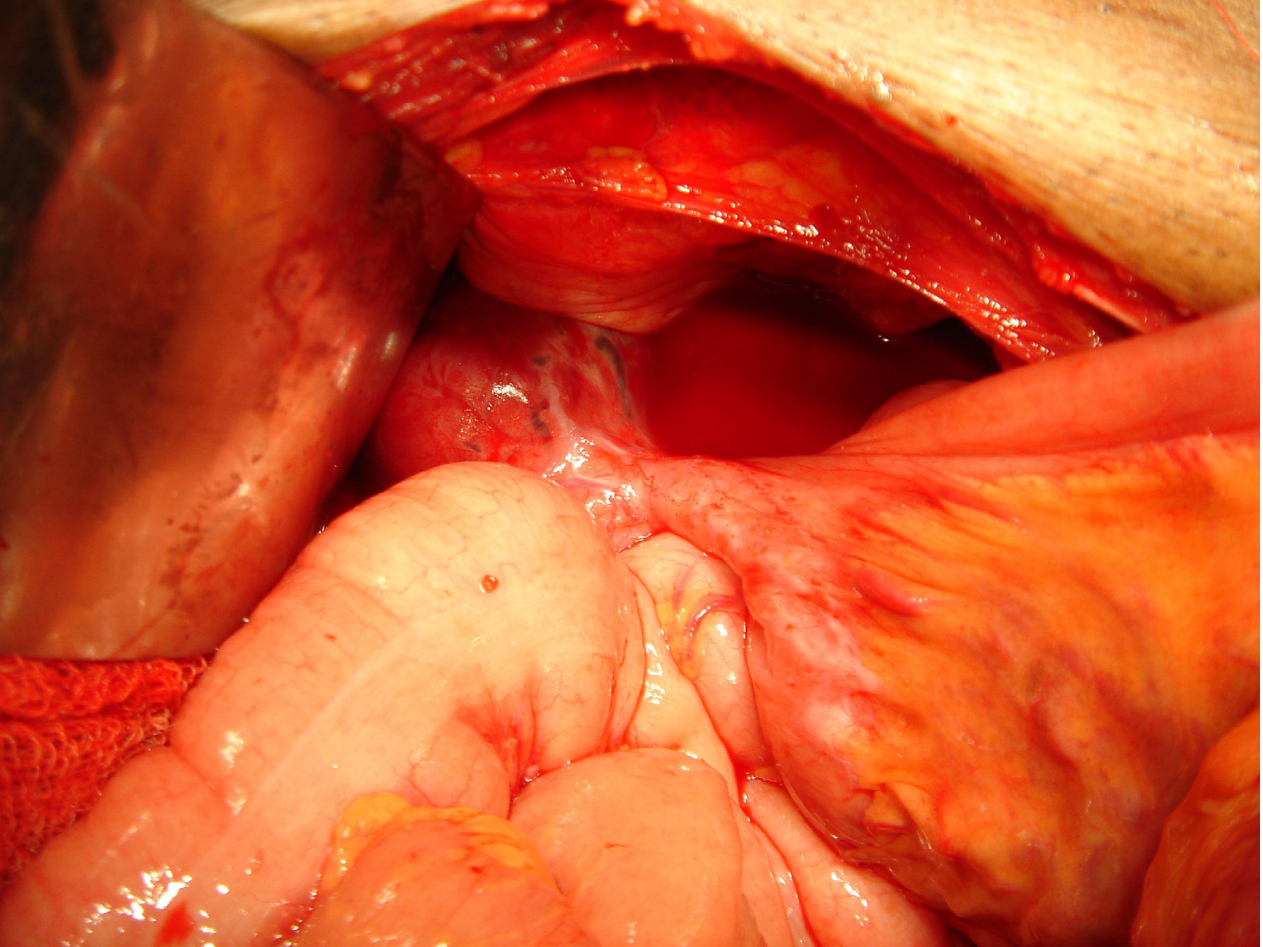
Intraoperative picture showing tumour arising from Meckel's diverticulum; Tumour was located in Meckel's diverticulum and adherent to wall of urinary bladder.

**Figure 3:**
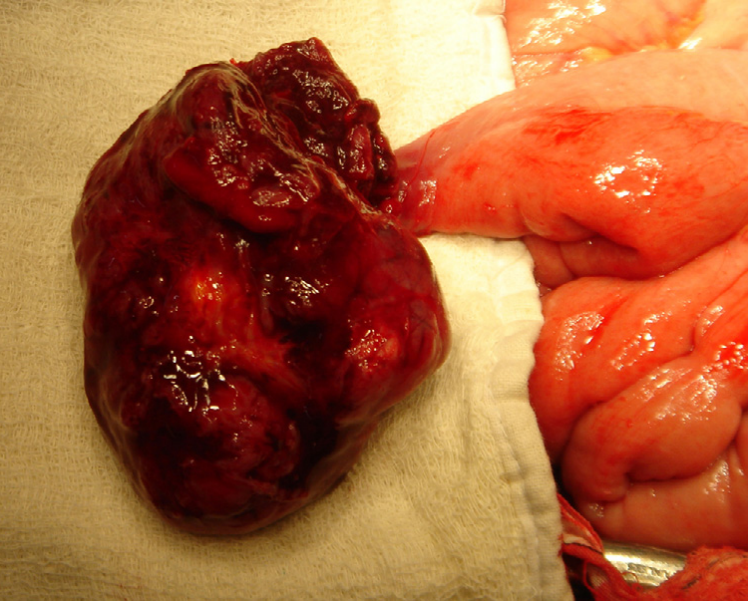
Intraoperative picture showing tumour and Meckel's diverticulum; Picture of Meckel's diverticulum with part of tumour, which was delivered out.

Sixty-five year old gentle man presented with constipation for 4 months and bleeding per rectum for one month. Physical examination was unremarkable. Ultrasonography of the abdomen revealed 6 × 9 cms exophytic hypoechoic lesion in pelvis near sigmoid colon. Barium enema study was normal. Colonoscopy showed colitis from anal canal up to 20 cms. Rest of Colon was normal up to Caecum. Contrast enhanced computerized tomography (CT) scan showed lobulated mass lesion in pelvis posteriosuperior to the urinary bladder compressing anterior wall of sigmoid colon. CT scan picture was suggestive of soft tissue tumour in close relation to sigmoid colon or sigmoid mesentery (figure 1), a diagnosis of small bowel tumor compressing sigmoid colon was made. An exploratory laparotomy was done. On laparotomy, the lobulated tumour seems to be arising from an ileal diverticulum, which was very short in length and situated 50 cms from ileocaecal valve. So diagnosis was soft tissue tumour arising from Meckel's diverticulum. The tumour was adherent to sigmoid colon and part of wall of urinary bladder (figure 2, 3). There was no evidence of distant spread. Tumour was excised with 3 cm of ileum on either side. End to end anastomosis was done in two layers. Involved area of anterior wall of sigmoid colon was also excised and the defect was closed transversally. Adherent part of the urinary bladder musculature was resected but the bladder mucosa was intact. Postoperative period was uneventful. Postoperatively urinary catheter was retained for 10 days.

**Figure 4:**
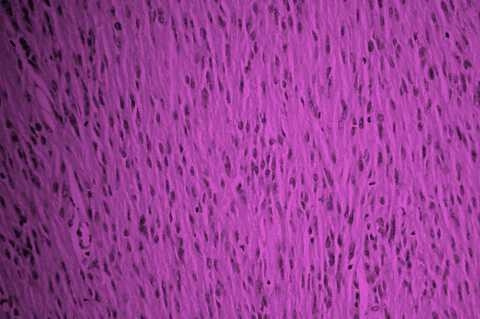
High power view of the histopathology slide; Tumour cells were pleomorphic and 2–3 mitosis were present in 50 high power fields.

**Figure 5:**
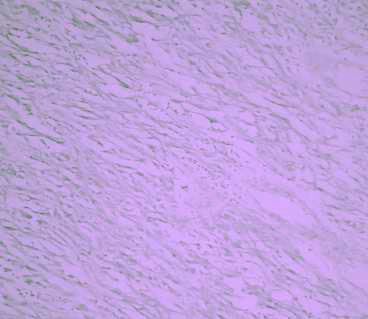
Histopathology slide after Immunostaining for C-Kit; Tumour cells show positivity after C-Kit staining, which suggests GIST.

The pathology report was gastrointestinal stromal tumour (GIST) arising from Meckel's diverticulum. The tumour cells were pleomorphic and 2–3 mitosis were present in 50 high power fields (figure 4). All margins were negative. Immuno-hisochemistry showed positive reaction for vimentin and C kit (Figure 5). But desmin, actin, S100, and CD 34 were negative.

## Discussion

Meckel's diverticulum is the most commonly encountered congenital anomaly of the small intestine, occurring in approximately 2% of the population [1,2]. Meckel's diverticulum is located on the antimesentric border of the ileum approximately 45 to 60 cm proximal to the ileocecal valve and results from incomplete closure of the omphalomesentric, or viteline duct. An equal incidence is found among men and women. Heterotopic mucosa is present in Meckel's diverticulum, the most common of which is gastric mucosa (present in 50% of all Meckel's diverticula). Pancreatic mucosa is encountered in approximately 5% of diverticula. Similarly these diverticula may habour colonic mucosa.

The vast majority of Meckel's diverticula are incidentally discovered during autopsy, laparotomy, or barium studies. The most common clinical presentations of the Meckel's diverticulum are gastrointestinal bleeding (from chronic acid-induced ulcer in the ileum adjacent to a Meckel's diverticulum that contains gastric mucosa), intestinal obstruction, and diverticulitis. Incidence of tumours within the Meckel's diverticulm is 0.5 to 3.2% [4-6]. Most of them are commonly benign tumours like leiomyomas, angiomas, and lipomas. Malignant neoplasms include adenocarcinoma (which commonly originate from the gastric mucosa), sarcoma, carcinoid tumour and GIST.

GISTs are rare neoplasms which account for 0.1–1% of gastrointestinal malignancies. The term itself was first used in 1983 by Mazur and Clark [7] to identify a heterogeneous group of tumours, all of them histologically characterized by hyperplastic fused cells, not necessarily leiomuscular ones, but even neural ones. Gastrointestinal stromal tumours arise from the interstitial cells of Cajal, pace maker cells of Gastrointestinal tract [8].

GIST occurs predominantly in adults at a median age of 58 years. The majority of GISTs (60% to 70%) have been reported to arise in the stomach, whereas 20% to 30% originate in the small intestine, and less than 10% in the esophagus, colon and rectum. GISTs also occur in the extra-intestinal abdominopelvic sites such as the omentum, mesentery, or retroperitoneum [9-11]. GISTs arising from Meckel's diverticulum are extremely rare [12-16].

For many patients, the detection of GIST may be an incidental finding during evaluation of nonspecific symptoms. Symptoms tend to arise only when tumours reach a large size or are in critical anatomic location. Most symptomatic patients present with tumours larger than 5 cm in maximal dimension. Symptoms at presentation may include abdominal pain, abdominal mass, nausea, vomiting, anorexia, and weight loss. The vast majority of metastatic GISTs are located intraabdominal, either in the liver, in the omentum, or in the peritoneal cavity [9]. Metastatic spread to lymph nodes and to other regions via lymphatics is very rare.

CT is usually an adequate technology to diagnose tumours arising from Meckel's diverticulum as long as appropriate techniques for both noncontrast and intravenous contrast administrations are used [13]. Histopathologically, disease exhibits a wide variety of appearances with characteristics of either epitheloid (approximately 70%) or spindle cell histology (remaining 30%). Normally, the KIT protein serves as a transmembrane RTK; the CD117 antigen can be detected by immunohistochemical staining as marker for the presence of the KIT protein. With the use of sophiscated technology, it has become clear that KIT mutations can be noted in more than 90% of GIST cells.

CD34 expression is not specific for GIST, because it can also be noted in desmoid tumours, and approximately 60% to 70% of GIST lesions are positive for CD34 [17-19]. Expert analysis of the KIT and PDGFRA genotype may be useful to define with certainty the group of rare patients with CD117-negative GISTs in the future. GIST is considered to be potentially malignant tumours. [19].

Most reliable prognostic factors are the size of the primary tumour and the mitotic index which measure proliferative activity of the cells. Other prognostic factors are specific histologic subtypes (epitheloid vs. spindle cell), the degree of cellular pleomorphism and age of the patient. Recurrence and survival rates have also been reported to correlate with the location of the primary GIST lesion, with small bowel tumours showing a somewhat worse prognosis. The functional imaging in GISTs with FDG-PET can give additional information that can assist clinicians in the management of patients. Definitive surgery remains the mainstay of treatment for patients with localized, primary GIST.

## Conclusion

Neoplasms arising from Meckel's diverticulae are differential diagnoses in patients presenting with pelvic masses of bowel origin, in which imaging modalities doesn't pinpoint a definitive diagnosis. Eventhough it is not so common, one should keep in mind this differential diagnosis in pelvic masses of suspected bowel origin.

## Competing interests

The author(s) declare that they have no competing interests.

## Authors' contributions

**CK**: Drafted manuscript

**MA**: Helped in preparation of the manuscript.

**RCG **– Assisted surgery and did literature review.

**GG **– interpreted and correlated imaging data with text narrative.

**MH **– Helped in drafting the manuscript

**PT **– Reported prepared pathology slides and photos.

**KCK **– Conceived idea, organized case reporting.

All authors read and approved the final manuscript.
